# Cytokine and Adipokine Levels in Patients with Premalignant Oral Lesions or in Patients with Oral Cancer Who Did or Did Not Receive 1α,25-Dihydroxyvitamin D_3_ Treatment upon Cancer Diagnosis

**DOI:** 10.3390/cancers7030827

**Published:** 2015-06-25

**Authors:** M. Rita I. Young, Corinne Levingston, Sara D. Johnson

**Affiliations:** 1 Medical Research Service (151), Ralph H. Johnson Veterans Affairs Medical Center, 109 Bee Street, Charleston, SC 29401, USA; E-Mail: cos25@musc.edu; 2 Department of Otolaryngology, Head and Neck Surgery, Medical University of South Carolina, 135 Rutledge Avenue, Charleston, SC 29425, USA; E-Mail: johsad@musc.edu

**Keywords:** adipokines, cytokines, HNSCC, inflammation, oral cancer, premalignant lesions

## Abstract

Differences in levels of inflammation-modulating cytokines and adipokines in patients with premalignant oral lesions *versus* in patients that develop squamous cell carcinoma of the head and neck (HNSCC) were assessed. Also assessed was the impact of treating HNSCC patients with the immune regulatory mediator, 1α,25-dihydroxyvitamin D_3_ [1,25(OH)_2_D_3_], on modulators of inflammation. Compared to healthy controls, patients with premalignant oral lesions had increases in their systemic levels of the inflammatory cytokines IL-6 and IL-17, and increases in the adipokine, leptin. However, levels of these pro-inflammatory cytokines and adipokine were reduced in patients with HNSCC. Treatment of HNSCC patients with 1,25(OH)_2_D_3_ increased levels of each of the measured immune mediators. Levels of the anti-inflammatory adipokine, adiponectin, were shifted inversely with the levels of the pro-inflammatory cytokines and with leptin. These studies demonstrate heightened immune reactivity in patients with premalignant lesions, which wanes in patients with HNSCC, but which is restored by treatment with 1,25(OH)_2_D_3_.

## 1. Introduction

Both cytokines and adipokines that regulate inflammation have been associated with cancer risks or with cancer patient outcomes, and there have been suggestions of using inflammatory mediators as biomarkers for oral cancer [1,2,3,4,5,6]. While multiple studies have focused on assessing the impact of obesity-associated inflammatory adipokines on cancer risk [5,7,8], adipocytes also have an immune regulatory capacity outside of the obese state [9,10,11]. Adipocytes can contribute to inflammatory states through their capacity to produce a multitude of mediators including interleukin (IL)-6, IL-8, IL-10, tumor necrosis factor (TNF)-α and TNF-β [12,13,14,15,16,17]. In addition, adipocytes can produce immune and angiogenic adipokines such as leptin, and the anti-inflammatory and anti-angiogenic adipokines such as adiponectin [18,19].

The interrelationship between inflammation and cancer risk has been an intense area of study, but far less emphasis has been placed on determining how the levels of pro- or anti-inflammatory adipokines and cytokines are associated with cancer progression or with responses to immune modulatory treatments. During the course of tumor development and, consequently, treatment, the immunological status is not stagnant. The immunological responses that have typically been evaluated during the course of tumor development or treatment include Th1 anti-tumor responses as well as immune inhibitory arms of immune reactivity that include Treg, Th2, and the less mature myeloid-derived suppressor cells or the related CD34^+^ progenitor cells [20,21,22,23,24,25,26]. However, tumor-associated immune regulatory activities also include inflammatory cytokines as well as immune regulatory adipokines [14,18,19].

Head and neck squamous cell carcinoma (HNSCC) is an aggressive malignancy in patients as well as in animal models. Patients with premalignant oral lesions have an increased risk of developing HNSCC and, consequently, a variety of measures are being tested in patients and in animal models to prevent progression to cancer [27,28,29]. Clinical variables that predict development of cancer in patients with premalignant oral lesions include the location, with the highest risk sites being the floor of mouth, tongue and lip [27]. The thickness of the premalignant oral lesion has also been directly linked to the risk for HNSCC. There are suggestions of heightened immune reactivity within patients’ premalignant oral lesions and this has been supported in animal models showing increased immune reactivity within the premalignant oral lesions and regional lymph nodes but a waning of this reactivity when lesions have progressed to cancer [30,31].

Prominent within patients and animals with HNSCC is immune dysfunction [20,32,33,34]. Among the immune inhibitory mechanisms described to be induced by HNSCC are defects in immune cell maturation and increases in levels of immature immune inhibitory cells such as MDSC or CD34^+^ progenitor cells [34,35]. Therefore, one strategy to overcome this immune subversive process is to promote differentiation of these suppressive cells into mature cells lacking the suppressive activity. Our prior trials with HNSCC patients have shown that treatment with vitamin D metabolites reduces levels of immune inhibitory immature CD34^+^ cells, increase levels of mature dendritic cells, stimulates both intratumoral immune infiltration and peripheral blood immune reactivity, and prolongs the post-surgical period prior to tumor recurrence [26,35,36,37,38]. Studies with other cancer types have shown vitamin D supplementation improves survival of breast cancer patients, reduces prostate cancer progression, and reduces the risk of developing colorectal cancer [39,40,41]. While some of the anti-cancer effects of vitamin D metabolites have been attributed to immune modulation, other studies have attributed the anti-cancer effects to be through interference in cellular proliferation [26,42,43,44].

The present study was undertaken to assess how premalignant oral lesions and the development of HNSCC impact on inflammatory cytokine and adipokine levels, and to identify how treatment of HNSCC patients with the active vitamin D metabolite, 1,25(OH)_2_D_3_, alters levels of these immune mediators. The results of this study demonstrate a systemic pro-inflammatory cytokine and adipokine milieu in patients with premalignant oral lesions, which subsides in patients with HNSCC. However, the levels of pro-inflammatory mediators is restored upon treatment of HNSCC patients with 1,25(OH)_2_D_3_.

## 2. Results

### 2.1. Patient Population

Studies measured systemic levels of select inflammation-modulating adipokines and cytokines in: (i) control healthy subjects; (ii) patients with premalignant oral lesions, which represents a population at risk for developing HNSCC; (iii) newly diagnosed patients with histologically proved HNSCC; and (iv) newly diagnosed HNSCC patients treated with 1,25(OH)_2_D_3_ prior to receiving any other definitive cancer therapy. Treatment of HNSCC patients with 1,25(OH)_2_D_3_ has previously been shown to enhance immune reactivity and prolong post-surgical time to recurrence [26,35,36]. Only the HNSCC population received the 1,25(OH)_2_D_3_ treatment since the treatment aimed to target an immature immune inhibitory cell population that is not apparent in patients with premalignant oral lesions or in healthy subjects. Approximately 30 subjects were recruited into each of the four arms of the study. Shown in Table 1 are the distributions of the patient populations based on age, gender, body mass index (BMI) and tumor stage. The distributions by gender and age were similar for the four groups. Of importance to this study was that the BMI was comparable among each of the cohorts.

**Table 1:** Patient populations.

Subjects	Details	Control		Premalignant		HNSCC		HNSCC-VitD	
Age		59.0 ± 11.3		60.7 ± 9.7		56.6 ± 10.5		61.8 ± 10.0	
Gender	male	19 (63%)		18 (56%)		21 (68%)		19 (63%)	
	female	11 (37%)		14 (44%)		10 (32%)		11 (37%)	
BMI kg/m^2^		27.8 ± 9.3		25.9 ± 6.1		26.5 ± 7.7		26.8 ± 7.6	
Stage	T2					11	(35%)	8	(27%)
	T3					6	(19%)	9	(30%)
	T4					14	(45%)	13	(43%)
	N0-N1					19	(61%)	19	(63%)
	N2-N3					12	(39%)	11	(37%)

### 2.2. Changes in Adiponectin and Leptin Levels with Cancer Development

Our prior studies with patient premalignant oral lesion and HNSCC tissues, as well as with a carcinogen-induced mouse oral lesion and cancer model had shown the presence of inflammatory cytokines within premalignant lesion tissues and a decline in these inflammatory cytokines within HSNCC [30,35]. The present study expanded these prior studies by determining if there are alterations of not only inflammatory cytokines, but also immune-regulating adipokines in patients with premalignant oral lesions and with HNSCC, and whether treatment to restore immune competence of HNSCC patients with 1,25(OH)_2_D_3_ impacts systemically on these mediators.

**Figure 1:**
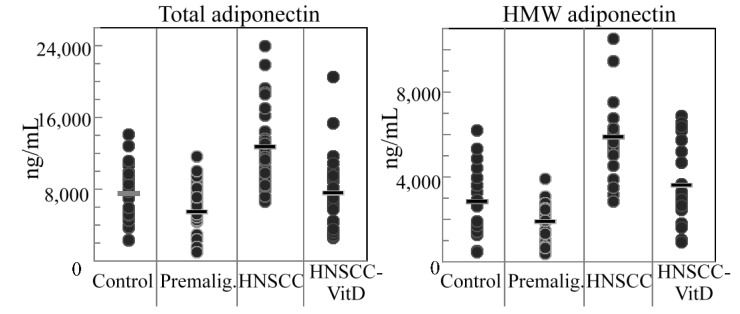
Plasma levels of adiponectin. Levels of total adiponectin (**left** panel) and high-molecular weight adiponectin (HMW; **right** panel) were measure in plasma of control healthy subjects, subjects with premalignant oral lesions (Premalig.), subjects with HNSCC or HNSCC subjects that were treated with 1,25(OH)_2_D_3_ (VitD).

Plasma levels of both total adiponectin and the multimeric high molecular weight (>300 kDa) adiponectin were measured. Total levels of adiponectin, which has anti-inflammatory activity [11,45], were reduced in patients with premalignant lesions compared to levels in subjects without lesions or cancer (Figure 1, left panel; Table 2). However, the levels increased in the untreated HNSCC cancer patients. Following treatment of HNSCC patients with 1,25(OH)_2_D_3_, adiponectin levels declined. Similar results were seen for high-molecular weight adiponectin in that levels were reduced in patients with premalignant oral lesion, increased in HNSCC patients, but then declined in HNSCC patients treated with 1,25(OH)_2_D_3_ (Figure 1, right panel; Table 2).

**Table 2:** Plasma levels of adiponectin and leptin, with significance of difference among groups.

Adipokine	Comparison	Control	Premalignant	HNSCC	HNSCC-VitD
Adiponectin	total, ng/mL	7562 ± 766	5497 ± 682	12,744 ± 1179	7610 ± 1007
	*p* *vs.* control		*p* < 0.01	*p* < 0.001	NS
	*p* *vs.* premalig.			*p* < 0.001	*p* < 0.02
	*p* *vs.* HNSCC				*p* < 0.001
	HMW, ng/mL	2931 ± 561	1902 ± 261	5897 ± 569	3620 ± 547
	*p* *vs.* control		*p* < 0.02	*p* < 0.001	NS
	*p* *vs.* premalig.			*P* < 0.001	*p* < 0.001
	*p* *vs.* HNSCC				*p* < 0.001
Leptin	ng/mL	8.48 ± 2.21	23.46 ± 4.87	8.22 ± 1.07	22.89 ± 4.05
	*p* *vs.* control		*p* < 0.001	NS	*p* < 0.001
	*p* *vs.* premalig.			*p* < 0.001	NS
	*p* *vs.* HNSCC				*p* < 0.001
Leptin/Adiponectin ratio, ×10^−3^		1.12	4.27	6.45	3.01

**Figure 2:**
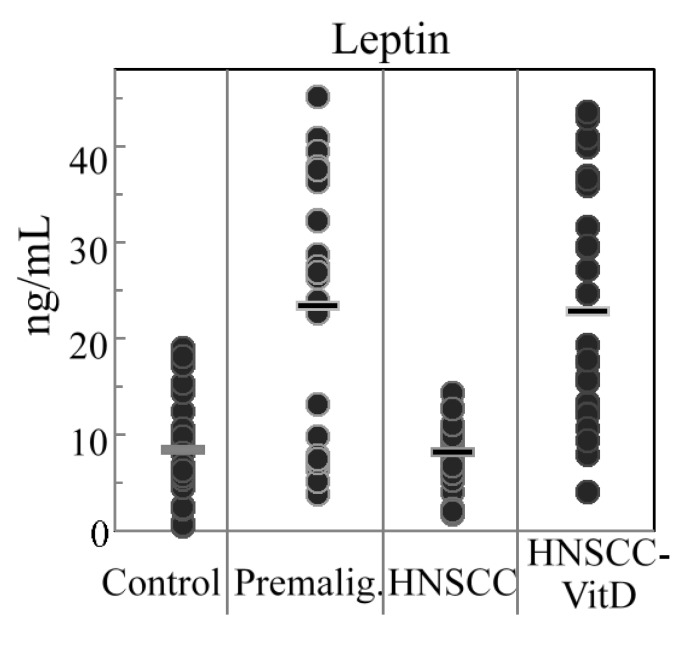
Plasma levels of leptin. Levels of leptin were measure in plasma of control healthy subjects, subjects with premalignant oral lesions, subjects with HNSCC or HNSCC subjects that were treated with 1,25(OH)_2_D_3_.

The results of leptin measurements were the inverse of those seen for adiponectin. Plasma levels of the immune-modulatory adipokine, leptin, [46,47], increased in patients with premalignant oral lesions but were reduced in the HNSCC patients (Figure 2, Table 2). HNSCC patients that were treated with 1,25(OH)_2_D_3_ had higher levels of leptin that were comparable to levels in patients with premalignant oral lesions. The combination of the results for adiponectin and leptin levels show an inverse relationship between leptin and adiponectin whereby systemic leptin levels are increased in patients with premalignant oral lesions and in 1,25(OH)_2_D_3_-treated HNSCC patients while adiponectin levels are decreased in these same patient populations and, inversely, adiponectin levels are increased and leptin levels decreased in in untreated HNSCC patients. These inverse changes are reflected by the more prominent differences in the adipokine levels when expressed as a ratio of leptin to adiponectin levels. The leptin to adiponectin ratio is 1.12 × 10^−3^ for control subjects, 4.27 × 10^−3^ for subjects with premalignant oral lesions, 0.65 × 10^−3^ for subjects with HNSCC and 3.01 × 10^−3^ for HNSCC subjects that received 1,25(OH)_2_D_3_ treatment.

### 2.3. Changes in Levels of the Inflammatory Cytokines IL-17 and IL-6 in Premalignant Lesion and HSNCC Tissues and Plasma

**Figure 3:**
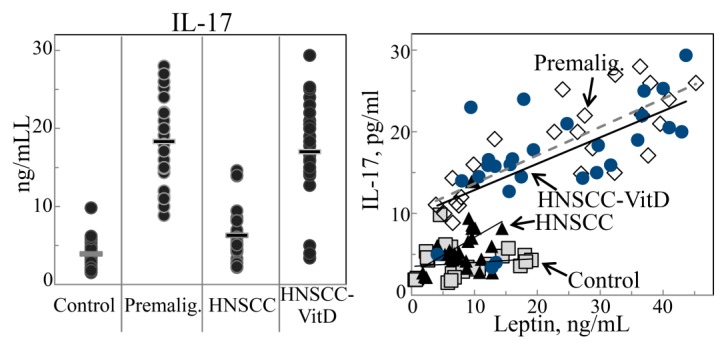
Plasma levels of IL-17 and association with increased leptin levels. Levels of IL-17 were measure in plasma of control healthy subjects, subjects with premalignant oral lesions, subjects with HNSCC or HNSCC subjects that were treated with 1,25(OH)_2_D_3_ (**left** panel). These plasma IL-17 levels of each patient were plotted against their plasma levels of leptin (**right** panel).

**Figure 4:**
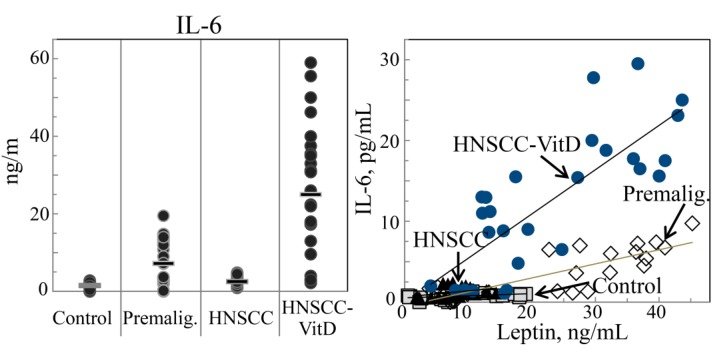
Plasma levels of IL-6 and association with increased leptin levels. Levels of IL-6 were measure in plasma of control healthy subjects, subjects with premalignant oral lesions, subjects with HNSCC or HNSCC subjects that were treated with 1,25(OH)_2_D_3_ (**left** panel). These plasma IL-6 levels of each patient were plotted against their plasma levels of leptin (**right** panel).

Our prior studies with a carcinogen-induced premalignant oral lesion model in which lesions progress to oral cancer had shown an increased inflammatory cytokine milieu within premalignant oral lesions and a decline in this cytokine composition once lesions progressed to oral cancer [31]. This prompted assessment of whether localized premalignant oral lesions impacted on patient systemic levels of inflammatory cytokines and whether this coincided with cytokine levels within the lesion or HNSCC tissues. Levels of the inflammatory cytokines IL-17 and IL-6 were relatively low in lysates of control normal oral tissues and the plasma of healthy controls without oral lesions or cancer (Table 3; Figure 3 and Figure 4, left panels). However, levels of these inflammatory cytokines were higher in premalignant oral lesion lysates and in plasma of patients with premalignant oral lesions. In patients with HNSCC, the HNSCC tissue and plasma levels of the inflammatory cytokines IL-17 and IL-6 were significantly lower than in lesion tissue and plasma of patients with premalignant oral lesions (Table 3; Figure 3 and Figure 4, left panels). Levels of the inflammatory cytokines IL-17 and IL-6 were also measured for HNSCC patients that were treated with 1,25(OH)_2_D_3_ to determine if the treatment, which had previously been shown to result in intratumoral immune infiltration [26,35,36,37], also modulated levels of inflammatory cytokines. Both IL-17 and IL-6 levels were increased within HNSCC tissues and plasma of HNSCC patients that received treatment with 1,25(OH)_2_D_3_ (Table 3; Figure 3 and Figure 4, left panels). These analyses showed that levels of inflammatory cytokines changed in parallel within tissue and plasma, with levels being increased in patients with premalignant oral lesions, lower in HNSCC patients and higher in 1,25(OH)_2_D_3_-treated HNSCC patients.

**Table 3:** Plasma and tissue levels of inflammatory cytokines, IL-17 and IL-6 (mean ± SEM).

Cytokine		Control	Premalignant	HNSCC	HNSCC-VitD
IL-17	tissue, pg/100 µg protein	1.2 ± 0.01	58.3 ± 10.7	10.1 ± 2.6	62.0 ± 13.3
	plasma, ng/mL	3.9 ± 1.8	18.3 ± 5.9	6.3 ± 3.5	17.0 ± 6.2
IL-6	tissue, pg/100 µg protein	78.2 ± 11.4	202.8 ± 41.9	121 ± 27.3	371.9 ± 46.8
	plasma, ng/mL	1.5 ± 0.3	7.2 ± 3.8	2.5 ± 1.2	24.9 ± 4.5

### 2.4. Changes in Levels of the Adipokine Leptin Coincide with Changes in Levels of the Inflammatory Cytokines IL-17 and IL-6

An analysis was conducted to determine if differences in levels of the inflammatory mediators IL-17 and IL-6 among the four groups of subjects coincided with differences of levels of the immune modulator leptin. IL-17 and leptin in each patient with premalignant oral lesions showed a significant correlation between the increased levels of leptin and the increased levels of IL-17 (Figure 3, right panel; *r* = 0.8049, *p* < 0.0001). A significant correlation was also seen between plasma levels of IL-6 within premalignant lesion patients and levels of leptin (Figure 4, right panel; *r* = 0.8230, *p* < 0.0001).

For HNSCC patients, the absolute levels of IL-17 were lower, but there remained a correlation between plasma levels of IL-17 and leptin (Figure 3, right panel; *r* = 0.4092, *p* = 0.0341). This association was not, however, as prominent as for levels of IL-17 and leptin in patients with premalignant oral lesions. Levels of IL-6 were sufficiently low in all HNSCC patients to preclude any statistical association between IL-6 and leptin levels (Figure 4, right panel).

In 1,25(OH)_2_D_3_-treated HNSCC patients, the increases in levels of IL-17 resembled the increases seen for leptin, with a strong correlation in levels of these two mediators (Figure 3, right panel; *r* = 0.6336, *p* = 0.00039). IL-6 levels were also increased in 1,25(OH)_2_D_3_-treated HNSCC patients, although the absolute plasma IL-6 levels varied among subjects over a broad range (Figure 4, left panel). Similar to that seen for the association between IL-17 and leptin, there was a significant relationship between levels of IL-6 and leptin in HNSCC patients that had received 1,25(OH)_2_D_3_ treatment (Figure 4, right panel; *r* = 0.790, *p* < 0.0001).

The associations between levels of the anti-inflammatory adipokine, adiponectin, and the inflammatory cytokines IL-17 and IL-6 were also analyzed. While there were trends toward negative associations between adiponectin and IL-17 levels, and between adiponectin and IL-6 levels, the extents of negative correlations were variable and not statistically significant (not shown).

## 3. Discussion

The present study identified changes in levels of the pro-inflammatory cytokines, IL-6 and IL-17, and the pro- and anti-inflammatory adipokines, leptin and adiponectin, in patients with premalignant oral lesions or in patients that have developed HNSCC that were either untreated or treated with 1,25(OH)_2_D_3_ to modulate immune reactivity. The results showed an inverse relationship between levels of the pro-inflammatory mediators (IL-6, IL-17 and leptin) and the anti-inflammatory mediator adiponectin, with systemic levels of pro-inflammatory mediators being increased in patients with premalignant oral lesions, but reduced in patients with HNSCC. HNSCC patients that were treated with 1,25(OH)_2_D_3_ had levels of these mediators that more closely reflected the pro-inflammatory profile seen in patients with premalignant oral lesions.

Obesity has previously been shown to regulate adiponectin levels, with increased obesity resulting in reduced levels of adiponectin. It is unlikely that obesity was a contributor to the observed reduction in adiponectin levels in patients with premalignant oral lesions or in HNSCC patients that were treated with 1,25(OH)_2_D_3_ as there were no significance differences in the body mass index among any of the four groups of patients. In addition, the possibility of circadian rhythmometry, which we and others have shown to impact on cytokine levels [48], was lessened by the collection of blood samples during the mid-afternoon.

The mechanisms underlying the increased pro-inflammatory cytokine and adipokine levels in patients with premalignant oral lesions are not known. The present study showed that the differences in the plasma levels of IL-17 and IL-6 among the subjects with lesions, HNSCC or HNSCC subjects that received 1,25(OH)_2_D_3_ treatment are analogous to the differences seen in the oral tissues from these patient groups. Such studies are consistent with the immunological analyses conducted in a mouse carcinogen-induced premalignant oral lesion model that progresses to oral cancer. In this model, the development of premalignant oral lesions was associated with increases in conventional lymphocytes expressing activation and memory markers within cervical lymph nodes compared to both control and HNSCC-bearing mice [31]. This would seem to reflect an immunological attempt to mount a dysplasia-clearing response, but which may ultimately be overcome. The possibility of a failed immune attempt is also supported by the cervical lymph node cells having a slight increase in T cell expression of the exhaustion marker KLRG1 compared to what is seen in control mice and in HNSCC-bearing mice. What was not assessed in this murine model is whether local immunological responses to lesions or HNSCC also extended to systemic changes, although the present study clearly shows systemic effects in levels of cytokines and adipokines.

The clinical and immunological inter-relationships between levels of immune-modulatory adipokines and cancer have been an area of intense investigation, but not yet defined. Increased levels of adiponectin, which has anti-inflammatory properties, was associated in a study of breast cancer patients with reduced disease-free survival [5]. Similar results of reduced overall survival were seen for patients with squamous cell esophageal cancer having increased serum levels of adiponectin [1]. These studies are consistent with our demonstration of increased adiponectin levels in untreated HNSCC patients and a reduced level in HNSCC patients treated with 1,25(OH)_2_D_3_, a treatment that we previously showed to enhance intratumoral immune infiltration and extend the period to post-surgical cancer recurrence [26,35,36]. The present study also showed increased serum levels of pro-inflammatory cytokines IL-6 and IL-17, and in levels of leptin in patients with premalignant oral lesions or in HNSCC patients treated with 1,25(OH)_2_D_3_, which is inverse to what is seen for adiponectin. *In vitro* studies into the mechanisms of the association between leptin and inflammation have shown the capacity of leptin to stimulate production of pro-inflammatory cytokines by cells such as keratinocyte and monocytes, thereby accentuating inflammation-associated diseases [46,49].

The results of the present study showed an interplay between cytokine and adipokine mediators associated with 1,25(OH)_2_D_3_ treatment of HNSCC patients. While these studies may suggest modulation of anti-tumor immune reactivities, they do not preclude direct anti-tumor effects of vitamin D analogs such as via pro-apoptotic signaling. Nevertheless, the present study extended our prior demonstration of intratumoral immune infiltration in response to 1,25(OH)_2_D_3_ to also demonstrating systemic increases in the immune mediators leptin, IL-6 and IL-17. [26,35,36]. What is paradoxical is the dichotomy of immunological effects that have been demonstrated for vitamin D metabolites, including in non-cancerous and cancerous instances. For example, 1,25(OH)_2_D_3_ suppresses the inflammatory effects of the Th1 responses in pulmonary tuberculosis, but simultaneously promotes macrophage bactericidal activity [50,51,52]. Vitamin D metabolites are protective against experimentally induced autoimmunity, and prevent dendritic, Tc1, and Th1 cell differentiation [50,53], which contrasts with results of treatment in HNSCC patients [26,35,36]. Studies with human peripheral blood mononuclear cells showed vitamin D to attenuate production of inflammatory cytokines in response to cancer cells [42]. Despite these studies showing immune tempering by vitamin D, increased overall survival has been shown for breast cancer patients with increased serum levels of vitamin D [5]. Studies using a hamster buccal pouch oral tumor model similarly showed clinical effectiveness of 1,25(OH)_2_D_3_ treatment [54]. Consequently, the inter-relationship between the vitamin D analogs and immune regulation seems to have differing responses depending on the immune status in the recipients of the treatment.

The present study showed clear shifts in levels of immune regulatory cytokines and adipokines in patients with pre-cancerous oral lesions, HNSCC cancer and in 1,25(OH)_2_D_3_- treated HNSCC patients and establishes the platform for additional studies in the future. Since this study showed changes in cytokines and adipokines between patients with premalignant oral lesions and HNSCC, a breakdown of HNSCC patients by stage could reveal when shifts occur and if they are occur early in tumor development or in late stages. Such analyses were limited in the present study since there would be insufficient statistical power if patients were stratified by stage. It is also important to note that the basal levels of vitamin D could influence the magnitude of the response to treatment with vitamin D metabolites, with a deficiency in levels possibly resulting in a greater impact of administered vitamin D metabolites. While the present study measured how treating HNSCC patients with the active metabolite 1,25(OH)_2_D_3_ modulates levels of inflammatory cytokines and adipokines, the study protocol has the limitation of not having measured either the basal pre-treatment or the post-treatment levels of vitamin D metabolites.

There are a number of limitations that are inherent to this study. First and foremost, the study was not designed to determine the underlying mechanisms that mediate the shifts in levels of immune regulatory cytokines and adipokines during either cancer development or 1,25(OH)_2_D_3_ treatment. Also not know are the sources of the cytokines and adipokines that contribute to the differences in levels among subjects with premalignant lesions or HNSCC or in HNSCC patients receiving 1,25(OH)_2_D_3_ treatment. The sources of the immune mediators could include the premalignant or malignant cells, the immune infiltrate or other stromal components that are impacted by the premalignant or HNSCC presence. For example, breast cancer cells have been shown to be capable of producing leptin to, in turn, impact on stromal cells and tumor-infiltrating macrophages [55]. Adding to the complexity of deciphering the mechanisms that contribute to differences in inflammatory states in patients with premalignant oral lesions or HNSCC are possible shifts in the level of expression for receptors for the inflammatory cytokines and adipokines. This has been seen in studies assessing expression of adiponectin receptors in Barrett’s esophagus and HNSCC, and expression of leptin receptors in oral cancer subjects [56,57]. Other than inflammatory impacts of leptin, a decline in the restriction of inflammation by the reduction in adiponectin levels could also increase the levels of inflammatory mediators. Not mutually exclusive is the possibility of immune attempts or the failures in immune reactivity toward premalignant oral lesions and, subsequently, shifts from an inflammatory to a subdued response as lesions progress to cancer, but then a restoration of these responses following 1,25(OH)_2_D_3_ treatment. Such mechanistic analyses will need to be conducted either *in vitro* and/or with animal models through studies that can be designed to interrupt components of the immunological cascades to better define causality of the responses and their overall clinical impact on disease.

## 4. Experimental Section

### 4.1. Research Subjects and Specimens

Recruitment of human subjects into this study was approved by the Institutional Review Board of record. Healthy control subjects, patients with premalignant oral lesions, or newly diagnosed patients with stage II–IV HNSCC who were being scheduled for definitive surgical treatment were eligible for enrollment into this study. Exclusion criteria included any prior anti-cancer treatments or treatment with anti-inflammatory drugs. The healthy control subjects had no evidence of premalignant lesions or malignant disease. Some of the HNSCC patients were randomly assigned to a 3-week course of treatment with 1,25(OH)_2_D_3_ for the period between diagnosis and surgical treatment (treatment schedule described below). Peripheral blood specimens from healthy control patients, patients with premalignant oral lesions, or HNSCC that were untreated or that received 1,25(OH)_2_D_3_ were collected for research purposes. In most cases, the blood specimens were collected during mid-afternoon encounters. Once collected, patient plasma was separated and stored frozen until used for cytokine or adipokine analyses.

In addition to the collection of blood specimens from the four groups of research subjects, premalignant oral tissues and HNSCC that were surgically excised as part of the patients’ treatment plan were collected for research purposes. Some of the HNSCC tissues from previously untreated patients were obtained from the Medical University of South Carolina Tissue Biorepository. Normal oral tissues were also obtained from the Biorepository. These latter tissues were deemed pathologically normal, and contained no microscopic evidence of malignant disease. Control, premalignant, and HNSCC tissues from patients were collected and homogenized by sonication. These samples were stored frozen until used for cytokine analyses.

### 4.2. 1,25(OH)_2_D_3_ Treatment

HNSCC patients that were randomized into the 1,25(OH)_2_D_3_ treatment arm received three cycles of 4 µg enteric 1,25(OH)_2_D_3_ for each of three sequential days followed by four days of no treatment. This treatment schedule has previously been shown to have minimal toxicity [26,35,36,58]. Nevertheless, serum calcium levels were measured weekly to monitor toxicity. At the conclusion of three cycles of this treatment, patients underwent resective HNSCC surgery. At the time of surgical treatment, peripheral blood samples and HNSCC tissues that was not needed for clinical purposes were collected from patients who were either treated or not treated with 1,25(OH)_2_D_3_. Plasma and tissue lysates from these peripheral blood specimens were then stored frozen until analysis.

### 4.3. Measurement of Immune Mediators

The levels of IL-6 and IL-17 (IL-17A) in plasma and tissue lysates were determined using a cytometric bead array cytokine kit from BD Biosciences (San Jose, CA, USA). Relative amounts of each cytokine were analyzed using FCAP Array software (Soft Flow, Inc., St. Louis Park, MN, USA). Plasma levels of leptin and levels of the multimeric high-molecular weight form of adiponectin (HMW; >300 kDa) were measured by ELISA using Quantikine kits from R&D Systems (Minneapolis, MN, USA). Total plasma adiponectin levels were measured with the use of an ELISA kit from Millipore (Billerica, MA, USA).

### 4.4. Statistical Analysis

Data were reported using the mean as a measure of central tendency ± standard error of the mean. To compare one variable condition between groups, the Wilcoxon signed-rank test was used. Differences between any two groups were tested for significance by Student’s *t*-test. Correlation between variables was tested with the Pearson correlation coefficient. All statistical tests were 2-tailed and significance was defined by a *p* value of less than 0.05.

## 5. Conclusions

The present study showed clear shifts in levels of immune regulatory cytokines and adipokines in patients with pre-cancerous oral lesions, HNSCC cancer and in 1,25(OH)_2_D_3_- treated HNSCC patients and establishes the platform for additional studies in the future.
